# Restoration of pyrethroid susceptibility in a highly resistant *Aedes aegypti* population

**DOI:** 10.1098/rsbl.2018.0022

**Published:** 2018-06-13

**Authors:** Marissa K. Grossman, Valentin Uc-Puc, Julian Rodriguez, David J. Cutler, Levi T. Morran, Pablo Manrique-Saide, Gonzalo M. Vazquez-Prokopec

**Affiliations:** 1 Pennsylvania State University, University Park, PA, USA; 2 Universidad Autonoma de Yucatan, Merida, Yucatan, Mexico; 3 Emory University, Atlanta, GA, USA

**Keywords:** insecticide resistance, pyrethroids, *kdr*, *Aedes aegypti*

## Abstract

Insecticide resistance has evolved in disease vectors worldwide, creating the urgent need to either develop new control methods or restore insecticide susceptibility to regain use of existing tools. Here we show that phenotypic susceptibility can be restored in a highly resistant field-derived strain of *Aedes aegypti* in only 10 generations through rearing them in the absence of insecticide.

## 1. Introduction

Insecticide resistance is an increasing challenge for disease control [1]. Of particular concern is resistance to pyrethroid insecticides, given their widespread use and low mammalian toxicity [1]. Populations of *Aedes aegypti*, the main dengue, chikungunya and Zika vector, are exhibiting increasingly high levels of pyrethroid resistance, commonly measured by the knock-down resistance (*kdr)* mutations [2–4]. These point mutations in the *para*-orthologous sodium channel gene disrupt insecticide binding to the voltage-gated sodium channels [5]. Two *kdr* mutations strongly associated with pyrethroid resistance in *Ae. aegypti* are the phenylalanine to cysteine mutation in 1534 (F1534C) and the valine to isoleucine mutation in 1016 (V1016I) [6,7].

Despite strong insecticide selection pressure, polymorphism is maintained at the *kdr* locus in *Ae. aegypti* field populations at fine spatial scales [8–11]. Such polymorphism may be indicative of a fitness cost to the mutations in the absence of insecticide. While fitness costs in resistant strains of *Ae. aegypti* have been demonstrated [12–14], there is limited empirical evidence demonstrating restoration of pyrethroid susceptibility without insecticide pressure. Instead, studies tend to focus on selection towards resistance, describing increases in both *kdr* allele frequencies with pyrethroid exposure [13,14] along with reductions in larval viability [12]. Here, we use semi-natural experiments to assess whether susceptibility can be restored in a resistant strain of *Ae. aegypti*.

## 2. Methods

### (a) Experimental design

We reared a field-derived population of *Ae. aegypti* in insect rearing tents, measuring W60 cm × D60 cm × H60 cm (BugDorm-2120F, MegaView Science), under two treatments: with insecticide and without insecticide. To incorporate insecticide, we covered two sides of the tent, approximately 3600 cm^2^ each, with Pramex™ Long Lasting Insecticidal Nets (MGK) containing Olyset™ Technology with 2% permethrin only. We replicated each treatment five times; all replicates were conducted simultaneously inside an uninhabited room in a typical house in Merida, Mexico to simulate semi-natural conditions.

For each replicate, we placed a 2 l white bucket containing 1 l of domestic water and 500–800 eggs into an experimental tent. We fed larvae bovine liver powder (MP Biomedicals) *ad libitum* to minimize effects of larval competition. A 10% sucrose solution was provided to adults daily. Emerged females were blood-fed once a week for two weeks by a human volunteer who placed his or her arm directly inside the cage. Females were allowed to lay eggs into black oviposition cups and each week eggs were removed from the tent to dry. After two weeks, all remaining eggs and adults were removed from the tent. Eggs were left to dry for one day, and then 500–800 were selected at random and placed into a clean 2 l bucket to start the next generation. Surviving adult mosquitoes were euthanized in a −20°C freezer. Fifteen female and 15 male surviving adults from each replicate were selected at random for *kdr* genotyping. This process was repeated for 10 generations (1 year). After generation 10, eggs from tents with insecticide were placed into tents without insecticide, and vice versa, to reverse selection pressure.

### (b) Strain characterization

Source *Ae. aegypti* were F1 from eggs collected in Merida, Mexico. The frequency of I1016 and C1534 alleles in the population was 0.595 and 0.937 respectively, and the population exhibited 13.7% knock-down to permethrin according to CDC bioassay protocols [15].

### (c) Resistance assays

At generations F0 and F10, we conducted CDC bottle bioassays (15) on 100 females per experimental replicate (four bottle replicates of 25 mosquitoes) to test for phenotypic resistance to technical grade permethrin at 15 µg ml^−1^. A susceptible reference strain (Cienega de Flores) and an acetone-coated bottle were used as controls. The proportion of mosquitoes knocked-down at 30 min was recorded, and a Welch two-sample *t*-test (used for unequal variance between groups) assessed the difference in knock-down proportions between treatments. Allele-specific real-time PCR determined the *kdr* genotypes for generations 1, 3, 7 and 10 following protocols outlined in Saavedra-Rodriguez *et al*. [7] for F1534C and Yanola *et al*. [16] for V1016I.

### (d) Analysis

These mutations are close on the chromosome, approximately 44.5 kb apart [17], so we calculated linkage disequilibrium at each generation and for each replicate (see electronic supplementary material for equations) [18]. Using the maximum-likelihood estimation of linkage disequilibrium, *D*, we estimated haplotype frequencies for each generation and each replicate. To calculate fitness of each haplotype, we aggregated all replicates from each generation to increase sample size and calculated overall haplotype frequency. In the insecticide treatment, the fitness of each haplotype was calculated as the average haplotype frequency with insecticide divided by the average haplotype frequency without insecticide. We estimated the fitness of each haplotype without insecticide by dividing the average haplotype frequency at generation F10 by the average haplotype frequency at generation F1 without insecticide. Relative fitness was calculated by normalizing each haplotype to the haplotype with the highest fitness in the treatment, which assumes that the haplotype with the highest frequency carries the highest fitness.

## 3. Results

### (a) Phenotypic resistance

**Figure 1.**
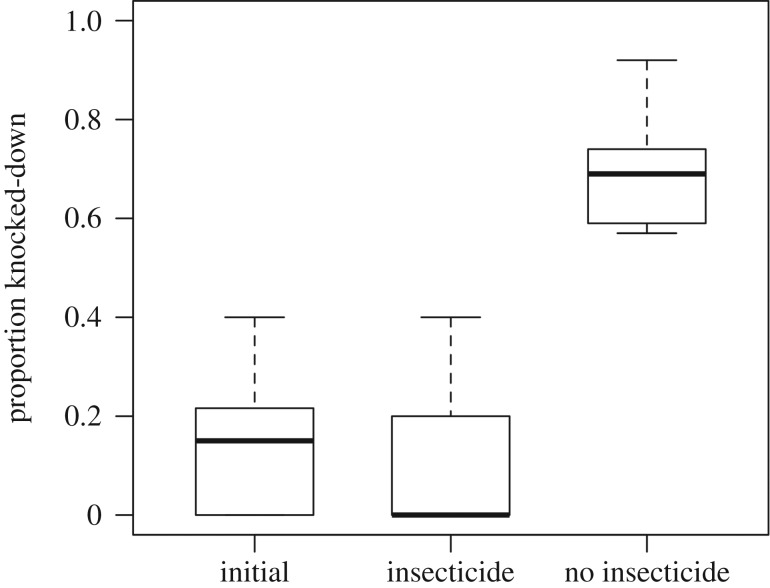
Phenotypic resistance. The proportion knocked-down at the diagnostic time of 30 min for the initial population (F0) and the two treatments at F10.

The mean proportion of knocked-down mosquitoes in the initial population (F0) was 0.14 ± 0.13, remaining unchanged over the course of 10 generations with insecticide exposure, ending with a knock-down proportion of 0.12 ± 0.17 (figure 1). However, the treatment without insecticide was 5.8 times more susceptible, displaying a knock-down proportion of 0.70 ± 0.14 (Welch *t*-test; *t* = −5.72, d.f. = 7.6, p-value ≤ 0.001). Additionally, 100% mortality was observed in F11 adults originating from no-insecticide treatments when placed into insecticide treatments; adults from F11 originating from insecticide treatments survived when placed into non-insecticide tents.

### (b) *kdr* haplotype frequencies

**Figure 2.**
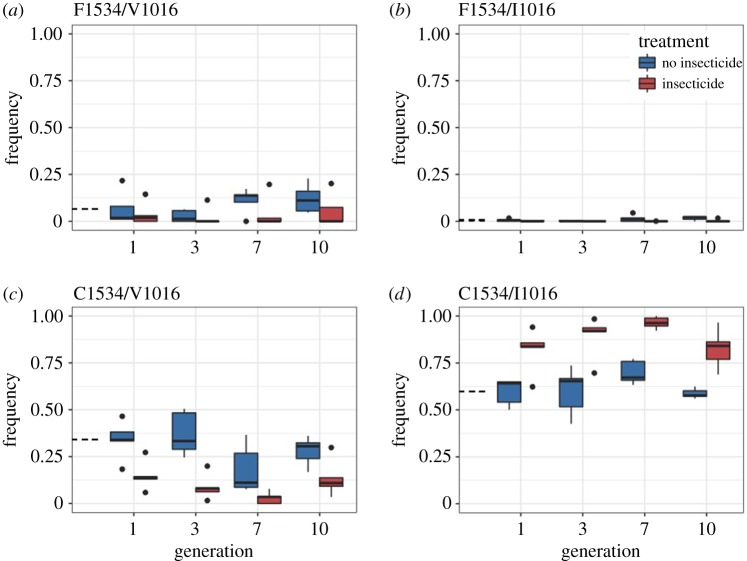
(*a*–*d*) Estimated haplotype frequencies over time in both the insecticide and non-insecticide treatments. Dotted lines next to the *y*-axis indicate initial frequencies. Sample sizes for the non-insecticide treatment are as follows: 141 for F1 and F3, 139 for F7, and 146 for F10; sample sizes for the insecticide treatment are: 105 for F1, 129 for F3, 148 for F7, and 167 for F10. (Online version in colour.)

The frequency of the wild-type haplotype, F1534/V1016, remained low and constant across 10 generations in the insecticide treatment (figure 2). However, without insecticide, the frequency increased from 0.07 ± 0.04 to 0.12 ± 0.03 (*χ*^2^ = 3.4, *p* = 0.064). The F1534/I1016 haplotype was rare, remaining at a frequency close to zero for both treatments (0.007 ± 0.003 without insecticide and 0.001 ± 0.001 with insecticide). C1534/V1016 was significantly lower with insecticide than without in both F1 (*χ*^2^ = 20.4, *p* < 0.001) and F10 (*χ*^2^ = 17.8, *p* < 0.001), and also significantly decreased from an initial frequency of 0.34 ± 0.05 to 0.14 ± 0.10 in F10 (*χ*^2^ = 32.1, *p* < 0.001). The frequency of the double-mutant haplotype, C1534/I1016, was significantly higher with insecticide than without (at F10: *χ*^2^ = 41.8, *p* < 0.001), and also significantly increased over time in the insecticide treatment (figure 2; *χ*^2^ = 37.0, *p* < 0.001). Fitness for each haplotype is shown in table 1.

**Table 1.** Fitness of each haplotype for both treatments. The estimated frequency of F1534/I1016 was approximately zero, and therefore we cannot estimate its fitness. Relative fitness was calculated by normalizing each haplotype to the haplotype with the highest fitness in the treatment.

treatment		F1534/V1016	F1534/I1016	C1534/V1016	C1534/I1016
insecticide	fitness	0.52	no data	0.32	1.41
	relative fitness	0.37		0.23	1
no insecticide	fitness	1.74	no data	0.83	0.98
	relative fitness	1		0.48	0.57

## 4. Discussion

We found a significant shift towards phenotypic susceptibility in populations without insecticide pressure. While populations did not meet the criterion for susceptibility defined by the World Health Organization, which is less than 90% knock-down [19], the significant loss of resistance in only 10 generations suggests that a longer duration without insecticide could drive populations below the susceptibility threshold. Despite a reduction in phenotypic susceptibility, the double-mutant haplotype (C1534/I1016) did not change significantly over time without insecticide. This begs the question: how do *kdr* mutations contribute to shaping phenotypic pyrethroid resistance and what other loci are responsible?

Both the I1016 and C1534 *kdr* mutations are well established in the literature as significantly associated with pyrethroid resistance [6,7,16,20,21], yet *kdr* is not the only genetic mechanism underlying resistance. Saavedra-Rodriguez *et al*. [22] found that while I1016 was the largest contributor to variance in *kdr* phenotype (approx. 58.6%), loci involved with metabolic detoxification of insecticide were also responsible. Additionally, an overexpression of mixed-function oxidases has been associated with deltamethrin-selected *Ae. aegypti* [14] and an upregulation of cytochrome *P*_450_ genes in permethrin-selected strains [23]. The lack of a strong association between *kdr* alleles and phenotypic resistance in our data suggests that metabolic resistance may be driving phenotype in our populations.

The relative fitness estimates of the haplotypes with and without insecticide give insight into the individual allele fitness, providing evidence of a fitness cost to C1534. Without insecticide, the wild-type haplotype, F1534/V1016, had the highest fitness. The C1534/V1016 haplotype, however, only had a fitness of one-half that of the wild-type haplotype, indicating a cost to C1534. Additionally, the absence of the F1534/I1016 haplotype indicates that either (1) I1016 is costly, or (2) the mutations are sequential in nature, with I1016 occurring only after the presence of C1534. Vera-Maloof *et al*. [24] also found a near absence of the F1534/I1016 haplotype in a linkage disequilibrium analysis of *kdr* mutations in field-caught Mexican *Ae. aegypti*, and similarly concluded that the mutations are likely evolved sequentially.

Overall, our results give compelling evidence of a fitness cost to the C1534 mutation and show that susceptibility can be restored in a highly permethrin-resistant *Ae. aegypti* population in the absence of insecticide. Only two other studies have evaluated the loss of pyrethroid resistance in *Ae. aegypti*. Both found a change after 15 generations: one quantified a decrease in I1016 frequency from 0.75 to 0.20 [13], yet did not evaluate phenotype, and the other found that the phenotype of a formerly permethrin-resistant population approached that of the susceptible strain [25]. It is important to note that *kdr* mutations are recessive to wild-type alleles, conferring resistance in the homozygous state [1]. Even when resistance is lost, *kdr* mutations may still be present in heterozygotes and could be selected for given a subsequent increase in insecticide pressure.

Future studies should evaluate the loss of resistance in field populations, as they are subject to ecological forces such as density-dependent competition [26] and environmental variation [27] that may modify the strength of selection for resistance mutations. Even though this study was conducted under laboratory conditions, the results support a vector control strategy that rotates chemicals in time and/or space, providing areas where resistant populations can revert to susceptibility while still using effective chemicals to suppress overall population abundance [28].

## Supplementary Material

SI Text: Methods Details

## Data accessibility

Data are publically available in the Dryad Digital Repository: (http://dx.doi.org/10.5061/dryad.33n9p). [29]
