# Supplementary material for: Restoration of pyrethroid susceptibility in a highly resistant Aedes aegypti population
Source: Biol Lett. 2018 Jun 13;14(6):20180022. doi: 10.1098/rsbl.2018.0022 (PMC6030600; doi:10.1098/rsbl.2018.0022)
Supplement: SI Text: Methods Details [file rsbl20180022supp1.docx]

**Supplementary Information**

**Restoration of pyrethroid susceptibility in a highly resistant *Aedes aegypti* population**

*Marissa K. Grossman^1^, Valentin Uc-Puc^2^, Julian Rodriguez^3^, David J. Cutler^3^, Levi T. Morran^3^, Pablo Manrique-Saide^2^, Gonzalo M. Vazquez-Prokopec^3^

*Haplotype frequency calculations*

We calculated allele frequencies at the 1534 and 1016 *kdr* loci for each replicate at generations F1, F3, F7, and F10. We assigned 1534 locus *A*, defining $p_{A1}$ as the frequency of F1534 (the susceptible allele), and 1016 was assigned locus *B*, with $p_{B1}$ as the frequency of V1016 (the susceptible allele). To look for signatures of selection, we conducted a test of Hardy-Weinberg Equilibrium (HWE) for each locus at each generation by calculating the inbreeding coefficient, *F* (using 1534 as an example):

$$F=1-\frac{obs(A_{1}A_{2})}{2p_{A1}p_{A2}}$$

When *F*=0, genotype frequencies are as expected under HWE; if 0<*F*≤1, there is an excess of homozygotes, and when F<0, there is an excess of heterozygotes (1). The quantity *nF*^2^ follows a Chi-square distribution with one degree of freedom, where n is the number of individuals.

Because these loci are physically close on the chromosome, we calculated linkage disequilibrium between them at each generation and for each replicate. The maximum likelihood estimation of linkage disequilibrium, *D*, is:

$D=\frac{1}{n}n_{A1B1}-2p_{A1}p_{B1}$ (2)

where *n* is the number of individuals and the digenic count, $n_{A1B1}$ , of *A_1_B_1_* + *A_1_*|*B_1_* is the following sum of genotype counts:

$n_{A1B1}= 2\left( A_{1}A_{1}B_{1}B_{1} \right)+ A_{1}A_{1}B_{1}B_{2}+A_{1}A_{2}B_{1}B_{1}+\frac{1}{2}(A_{1}A_{2}B_{1}B_{2})$ (2)

Using *D,* we estimated haplotype frequencies for each generation and each replicate:

$$A_{1}B_{1}=D+p_{A1}p_{B1}$$

$$A_{1}B_{2}=p_{A1}p_{B2}-D$$

$$A_{2}B_{1}=p_{A2}p_{B1}-D$$

$$A_{2}B_{2}=D+p_{A2}p_{B2}$$

To calculate the fitness of each haplotype, we aggregated all replicates from each generation to increase sample size for a more precise estimation and calculated overall haplotype frequency. In the insecticide treatment, the fitness of each haplotype compared to the control was calculated as simply the average haplotype frequency with insecticide divided by the average haplotype frequency in the control. For the control treatment, we estimated the fitness of each haplotype in the absence of insecticide over time by dividing the average haplotype frequency at generation F10 by the average haplotype frequency at generation F1. The fitness for each haplotype within a treatment was then normalized to the haplotype with the highest fitness, creating a measure of relative fitness.

1. Gillespie JH. Population Genetics. Second ed. Baltimore, MD: John Hopkins University Press; 2004.

2. Weir BS. Genetic Data Analysis II: Methods for Discrete Population Genetic Data: Sinauer Associates; 1996.
